# Consumption of dietary supplements to support weight reduction in adults according to sociodemographic background, body mass index, waist-hip ratio, body fat and physical activity

**DOI:** 10.1186/s41043-019-0191-3

**Published:** 2019-11-05

**Authors:** Adrian Lubowiecki-Vikuk, Magdalena Król-Zielińska, Adam Kantanista

**Affiliations:** 1 Department of Consumer Behaviour Research, Collegium of Management and Finance, Warsaw School of Economics, Al. Niepodległości 162, 02-554 Warsaw, Poland; 2 Department of Physical Education and Lifelong Sports, Poznan University of Physical Education, Królowej Jadwigi 27/39, 61-871 Poznań, Poland

**Keywords:** Weight management, Exercise, Overweight, Obesity, Poland

## Abstract

**Background:**

The aim of this study was to analyse the use of dietary supplements to support weight reduction (DSSWR) in adults according to sociodemographic background, body mass index (BMI), waist-hip ratio (WHR), body fat percentage (%BF) and level of physical activity (PA).

**Method:**

Participants (*n* = 1130) were recruited from a region of Poland with a high rate of adult overweight and obesity. Based on anthropometric data, BMI and WHR were calculated. %BF was assessed using a bioimpedance method. To examine the association between DSSWR use and sociodemographic factors, BMI, WHR, %BF and PA multiple logistic regression were conducted.

**Results:**

The rate of DSSWR use in the group studied was high (69.5%). A higher proportion of women, individuals aged 18–35 years, those who had completed higher education, those who did not report financial status as “poor”, with a BMI < 18.5, normal %BF and individuals with a high level of PA used DSSWR. In complete case analysis (*n* = 1108), primarily financial status reported as “good” (OR = 2.18, 95% CI: 1.69, 2.81) or “hard to say” (OR = 2.41, 95% CI: 1.86, 3.12) (vs. “poor”) and female sex (OR = 2.59, 95% CI: 2.17, 3.08) were associated with DSSWR intake.

**Conclusion:**

It seems that primarily financial status and sex, but also age, education, and level of PA, have significance in DSSWR use in adults and may be considered when developing appropriate strategies for body weight management and health promotion.

## Background

A well-balanced diet, combined with physical activity (PA), seems to be the appropriate method of maintaining a healthy body weight [1]. However, many adults use dietary supplements, considering them to be part of a well-rounded approach to body weight management [2]. Despite the lack of compelling evidence for any significant impact of supplement use on weight reduction [3–7], supplements are becoming increasingly popular [8–11]. In the USA, more than 60% of the adult [12] and, in Australia, more than 70% of the university population [13] used a dietary supplement. The interest in dietary supplements supporting weight reduction (DSSWR) has been noted in Poland [14, 15], and also in other countries [10, 16].

The use of various dietary supplements may be associated with the nature of postmodern society, with its passive lifestyles, excessive consumption and medicalisation of the body, and pressure on individuals to maintain a healthy and attractive body [10, 17–19]. The marketing activities of the pharmaceutical industry are also a factor of increase in dietary supplements use [20], despite known cases of the use of poor-quality production processes or the contamination of some supplements with prohibited substances [21]. Despite concerns of efficacy and safety, supplements are still used in weight management [7]. These supplements are advertised as requiring less effort than diet and exercise, with claims of effectiveness, are often cheap and are commonly available.

PA may be the cheapest way to reduce body weight, but is time-consuming and requires physical effort. Only the appropriate type, frequency and intensity of PA can reduce body weight in people of different ages [22, 23]. The use of DSSWR may be considered either a substitute for, or complement to, PA and a reduction of caloric intake. It must, however, be noted that using DSSWR is less demanding than maintaining a diet and increasing PA [24].

The use of dietary supplements may be affected by demographic and sociocultural factors [12, 25, 26]. Most users of dietary supplements are female, older [27], and college- or university-educated. According to Pillitteri et al. [10], the use of DSSWR was more common among women, younger adults and individuals with less education and lower incomes. The results are inconclusive, however, because, according to other studies [28–31], dietary supplement use was positively associated with education, income and age. In addition, some studies have indicated a growing number of supplement users who were also physically active [29, 32, 33], especially among men and individuals above the age of 45 years [27]. In these studies, though, the types and level of PA were not reliably measured. Therefore, it seems that the set of determinants of DSSWR use is not yet fully known.

Efforts to reduce body weight concern, in particular, people who are overweight or obese. This phenomenon is growing because the prevalence of obesity all over the world has increased from 3.2% in 1975 to 10.8% in 2014 in men, and from 6.4 to 14.9% in women [34]. In Poland (population is over 38.4 million people), the trend of obesity prevalence in adults is also adverse, and its growth since 1975 is one of the largest in the world. In men, 3.6 million and, in women, 4.3 million are obese [34]. Weight management in underweight individuals is also prevalent [35], but the studies are limited. In Poland, 60.6% of underweight and 61.7% of overweight adults have attempted to lose weight during the last 6 months, and many of them used slimming preparations [11]. According to Kozłowska and Pol [36], the main reasons for using DSSWR in adults were aesthetic concerns (63.8%), low self-esteem (48.3%) and the fashion for slim body (29.3%). People with excessive or deficient body weight are willing to change their body status because they may be dissatisfied with their weight and appearance. Body dissatisfaction has been observed in many women and men [37, 38]. Women usually wish to be thinner [39, 40]. Dissatisfaction with body fat percentage (%BF) and muscle tone is common in adult men [41], as well as the desire to lose weight as they get older [42].

Identification practice in DSSWR may be helpful in designing appropriate health programmes for weight management in different groups of people. Additionally, monitoring the consumption of DSSWR in various social groups gives the opportunity to take action to raise awareness of the risks arising from their abuse.

Therefore, the aim of this study was to analyse the use of DSSWR in adult people, with regard to sociodemographic background, body mass index (BMI), waist-hip ratio (WHR), %BF and level of PA.

## Methods

### Participants and procedures

The study included 1130 participants (assumed error 3%, typical choice 95%), aged 18–70 years (mean = 43.4 years, standard deviation = 15.6 years). The participants were from Świętochłowice in the Silesian Voivodeship, a town with one of the three highest rates of adult obesity in Poland [43].

The study was performed by trained and supervised interviewers (with a degree in Pharmacy), according to a predetermined plan, between March and April 2017. Respondents were recruited via a public call (posters hung on advertising columns belonging to the town hall) for volunteers aged 18 years and above. The research was carried out in two stages: (1) measurement of body weight and height, calculation of BMI and WHR, then %BF evaluation, and (2) completion of a paper questionnaire regarding PA level, sociodemographic variables and DSSWR use. Anthropometric measurements were performed in a designated private location to ensure discretion and comfort.

### Use of dietary supplements supporting weight reduction, evaluation of sociodemographic characteristics and undertaking of physical activity

To evaluate the use of dietary supplements, the participants were asked whether they had used any DSSWR in the previous 3 months: “yes” or “no” answers were possible. They listed the trade name of supplements they had used. Next, it was decided whether the given products were DSSWR.

The sociodemographic characteristics of the respondents included sex (female, male), age (three categories were analysed: 18–35 years old, 36–60 years old, older than 60 years), marital status (married or unmarried) and education (primary, vocational, secondary, higher). Additionally, respondents were asked “What is your financial status?”. Response options were “good”, “poor” or “hard to say”. To evaluate the level of PA, the self-reported Polish version of the International Physical Activity Questionnaire—Short Form (IPAQ-SF) [44] was used. The IPAQ-SF was completed during face-to-face interview. Participants were asked to recall the type and duration of their physical activities in the last 7 days. Based on their data, the metabolic equivalent (MET) was calculated. The results were presented as an estimation of energy expenditure in metabolic equivalent (MET). The MET-min week^−1^ was calculated as follows: minutes of activity/day × days per week × MET value. From this continuous variable of total PA scores, the data were categorised according to the IPAQ scoring guidelines. Participants with a total PA of < 600 MET-min week^−1^ were classified in the “low” category, 600–2999 MET-min week^−1^ in the “moderate” category, and ≥ 3000 MET-min week^−1^ in the “high” category.

### BMI, WHR and %BF

Weight was measured using an Omron scale in light clothing (without shoes) to the nearest 0.5 kg. Height was measured with an anthropometer to the nearest 0.5 cm. The body height and weight of the participants were used to calculate BMI (kg/m^2^). BMI values below 18.5 indicated underweight, from 18.5 to 24.99 normal weight and 25 and over overweight. Waist circumferences were measured at the end of several consecutive natural breaths, at a level parallel to the floor, midpoint between the top of the iliac crest and the lower margin of the last palpable rib in the midaxillary line. Hip circumference was measured at a level parallel to the floor, at the largest circumference of the buttocks. The WHR was calculated by dividing waist circumference (in cm) by hip circumference (in cm). The recommendations for sex-specific cut-off points were 94 cm (men) and 80 cm (women) for increased disease risk and 102 cm (men) and 88 cm (women) for substantially increased risk. WHRs > 0.9 in men and > 0.85 in women denoted abdominal obesity [45].

An Omron Body Fat Analyzer model HBF-360 (Omron Healthcare, Inc., Vernon Hills, IL, USA) was used to measure body fat in men and women. The participants’ height, weight, age and sex were entered into the analyser. While standing with their feet slightly apart, the participant grasped the grip electrodes and held the analyser in front of their body, with arms fully extended and parallel to the floor. Each assessment took less than 1 min to complete. Although the bioimpedance method is not as accurate as, for example, dual-energy X-ray absorptiometry [46], according to Malavolti et al. [47], it is considered to be a valid, non-invasive, inexpensive method of determining total and regional body composition. The most frequently used %BF cut-off points for defining obesity (25% in men, 35% in women) were used [48, 49].

### Analysis of data

The examined variables were either nominal (sex, marital status, education, financial status and use of DSSWR) or categorised (age category, BMI status, WHR status, %BF content and PA level), and were presented by number and percentage distribution. First of all, the chi-square independence test was used to determine whether there were differences between the use and non-use of DSSWR (dependent, nominal, dichotomous variable) and each independent variable (sex, age, marital status, education, financial status, BMI status, WHR status, %BF content and PA level). Next, for all significant associations, one-way logistic regression analyses were conducted in order to investigate the association between the use of DSSWR (“yes” classification) and then statistically significant factors were included in multiple logistic regression. The analysis to examine the association between use of DSSWR and the predictors was done based on data from 1108 participants, because not all (1.95%) respondents completed all questions. The odds ratio (OR) was calculated with the confidence interval (95% CI) which allows one to determine whether this association is statistically significant. An area under the ROC curve (AUC) was calculated to assess the prediction quality of the multifactor model. The threshold for statistical significance for the inclusion of an independent variable in a multiple regression model was set at *p* value < 0.05. All calculations were performed using Statistica 13.0 (StatSoft, Inc.).

## Results

Detailed descriptive statistics of the examined variables are presented in Table 1. Out of all respondents, 69.5% declared they used DSSWR (Table 1).

**Table 1:** Descriptive statistics of variables among respondents divided into use and no use of dietary supplements supporting weight reduction (DSSWR), and differences between use of DSSWR and independent variables

Variables	All subjects		Use of DSSWR				*p* value
			Yes		No		
	*n*	%	*n*	%	*n*	%	
Sex							
Women	628	55.5	513	65.4	114	33.1	< 0.001
Men	502	44.5	272	34.6	230	66.9	
Age category							
18–35 years old	422	37.4	319	40.6	103	29.9	< 0.001
36–60 years old	477	42.2	338	43.1	138	40.2	
Older than 60 years	231	20.4	128	16.3	103	29.9	
Marital status							
Married	637	57.5	429	55.9	208	61.2	0.103
Unmarried	471	42.5	338	44.1	132	38.8	
Education							
Higher	263	23.4	228	29.2	34	10.0	< 0.001
Secondary	345	30.8	259	33.2	86	25.3	
Vocational	452	40.3	268	34.3	184	54.1	
Primary	62	5.5	26	3.3	36	10.6	
Financial status							
“Good”	687	61.3	536	68.6	151	44.5	< 0.001
“Hard to say”	275	24.6	206	26.4	69	20.4	
“Poor”	158	14.1	39	5.0	119	35.1	
BMI status							
Underweight	54	4.8	47	6.0	7	2.0	0.007
Normal	478	42.3	338	43.1	140	40.7	
Overweight	597	52.9	399	50.9	197	57.3	
WHR status							
Normal	967	86.4	677	86.9	289	85.3	0.458
Abdominal obesity	152	13.6	102	13.1	50	14.7	
%BF							
Normal	512	45.5	389	49.9	123	35.8	< 0.001
Above	613	54.5	391	50.1	221	64.2	
PA level							
High	293	25.9	238	30.3	54	15.7	< 0.001
Moderate	432	38.2	306	39.0	126	36.6	
Low	405	35.9	241	30.7	164	47.7	
Total		100	785	69.5	344	30.5	

Among those people using DSSWR, most were women (65.4%), aged 36–60 (43.1%), were in a relationship (55.9%), had a vocational education (34.3%), assessed their own financial situation as “good” (68.6%) and were overweight (50.9%), based on their BMIs. Furthermore, among people using DSSWR, the percentage share of people with normal or above normal %BF was equal, 30.3% undertook PA at a high level, 39% at a moderate level and 30.7% at a low level. Table 1 also presents the differences between the use or non-use of DSSWR and all independent variables. There were significant differences between the use of DSSWR and sex (*p* value < 0.001), age category (*p* value < 0.001), education (*p* value < 0.001), financial status (*p* value < 0.001), BMI status (*p* value = 0.007), %BF (*p* value < 0.001) and PA level (*p* value < 0.001).

The results of the one-way logistic regression and multiple logistic regression of the use of DSSWR for each significant independent variable are shown in Table 2.

**Table 2:** Results of one-way logistic regressions and multiple logistic regression analysis of use of dietary supplements supporting weight reduction (DSSWR)

Variables	Use of DSSWR				
	One-way logistic regressions		Multiple logistic regression		
	OR (95% CI)	*p* value	OR (95% CI)		*p* value
Sex					
Women	1.95 (1.71–2.23)	< 0.001	2.59 (2.17–3.08)		< 0.001
Age category					
18–35 years old	1.47 (1.22–1.76)	< 0.001	1.29 (1.03–1.61)		0.026
36–60 years old	1.16 (0.97–1.38)	0.096			
Education					
Higher	3.12 (2.30–4.25)	< 0.001	1.67 (1.14–2.44)		0.008
Secondary	1.40 (1.10–1.79)	0.007			
Financial status					
“Good”	2.34 (1.93–2.84)	< 0.001	2.41 (1.86–3.12)		< 0.001
“Hard to say”	1.97 (1.57–2.47)	< 0.001	2.18 (1.69–2.81)		< 0.001
BMI status					
Underweight	2.10 (1.23–3.59)	0.007			
%BF					
Normal	1.34 (1.17–1.52)	< 0.001			
PA level					
High	1.76 (1.41–2.19)	< 0.001	1.43 (1.11–1.86)		0.006

In women, an almost twice as high chance of using DSSWR was found (OR = 1.95, 95% CI 1.71, 2.23) compared to men. People aged 18–35 years had a one and a half times higher chance of using DSSWR (OR = 1.47, 95% CI 1.22, 1.76) than people from older age categories. In the group of people with higher education, there was a more than three times higher chance of using DSSWR (OR = 3.12, 95% CI 2.30, 4.25), and in the group of people with secondary education, the odds ratio was 1.4 (95% CI 1.10, 1.79). The “good” financial status of the respondents increased their chances of using DSSWR by more than twice (OR = 2.34, 95% CI 1.93, 2.84). A greater chance of using DSSWR was found among people who were underweight based on their BMI (OR = 2.10, 95% CI 1.23, 3.59), had normal %BF (OR = 1.34, 95% CI 1.17, 1.52) and undertook PA at a high level (OR = 1.76, 95% CI 1.41, 2.19).

In the final step, a multiple logistic regression was carried out. All relevant factors were inserted into the logistic regression model. According to the model, the use of DSSWR was predicted by five variables: financial status, sex, PA level, age category and education.

The use of DSSWR was explained first by financial status and sex. People who did not specify their financial situation or declared it to be “good” were over two times more likely to use DSSWR (OR = 2.41, 95% CI 1.86, 3.12 and OR = 2.18, 95% CI 1.69, 2.81, respectively). Women had a more than two and a half times greater chance of using DSSWR (OR = 2.59, 95% CI 2.17, 3.08). In addition, a greater chance of using DSSWR was found among people undertaking PA at a high level (OR = 1.43), from the youngest age category (OR = 1.29) and with higher education (OR = 1.67).

In order to determine the quality of the predictions for the use of DSSWR, based on the variables selected for the model, an AUC was also calculated and was found to be 0.821, which means that the model was characterised by good discrimination.

## Discussion

The aim of the study was to analyse the use of DSSWR in adults with regard to sociodemographic background, BMI, WHR, %BF and PA. The rate of DSSWR use was high (69.5%). The likelihood of using DSSWR was higher in women than in men, in individuals aged 18–35 than among older people, in those who had completed higher education than in the less educated, in those who declared with “good” and “hard to say” financial status than in those with “poor” financial status and in those performing a high level of PA than those doing moderate and low levels of PA.

Similar results were obtained by Pillitteri et al. [10], regarding sex (women vs. men) and age (25–34 years), but different regarding education and financial status. This may be due to the fact that the population they studied (*n* = 1444) mainly comprised individuals who had long and unsuccessfully tried to manage their body weight and had the wrong impressions about the supplements they were using [10]. Another profile of American DSSWR users (*n* = 9403) was performed by Blanck et al. [2]. They found that women were more likely to use DSSWR than men, especially when considering the age bracket (18–35 years) and average income. Among men, the likelihood of DSSWR use was higher in younger individuals (18–35 years) who had completed higher education. Our results show that the chances of using DSSWR among adults primarily increase with financial status, and are higher among women, which refers to earlier research by Blanck et al. [50] and Machado et al. [51]. The result highlights the global trend of consumerism and socio-cultural women’s attachment to the attractive appearance [17, 18]. Subsequently, the chances of using DSSWR increase in relation to young people and those with higher education, which can be explained by the competences (knowledge and skills) of consumers [52].

In our study, the likelihood of DSSWR use was particularly high in those who engaged in high PA levels, and decreased among those with low PA levels. It seems likely that the latter group may consider DSSWR to be a substitute for PA, while the former may use DSSWR to complement the PA and increase the effectiveness of their efforts to achieve the desired body shape. Associations between DSSWR use and PA have been demonstrated by Kofoed et al. [26], Blanck et al. [50] and Fassier et al. [53]. This suggests that PA may be treated as a complementary method to support the reduction of body weight (which is or is not excessive). Thompson and Thomas [27] indicated that an association between PA and DSSWR use was more commonly found among men choosing simple forms of PA, such as walking or cycling.

In our study, it was found that %BF and BMI were significantly associated with DSSWR use, while WHR did not have statistical significance. Ultimately, when using the multiple logistic regression model, these factors did not matter. So far, the impact of %BF on DSSWR use has been confirmed in metabolic studies, but only on specific products [54–56]. Regarding BMI, the evidence regarding the associations between DSSWR use and BMI is inconclusive [2, 4, 6, 10, 29, 36], and further investigations are warranted.

Analysing the association of DSSWR use with just a single factor may only provide explanatory results, rather than definitive ones, with regard to body weight management. Therefore, the inclusion of additional factors seems necessary. While estimating the likelihood of DSSWR use, PA levels should be considered, along with the subjects’ financial standing, sex, age and education. Our findings clearly indicate that DSSWR use rates are higher among people, who declared with “good” and “hard to say” financial status, especially young and educated women. Although the use of DSSWR is associated with risky health-related behaviours, as emphasised in the literature on the subject [3–7, 21, 57], it is interesting to note that DSSWR is used in combination with PA.

This study had several limitations. First, it only considered the current use of DSSWR among adults in the town of Świętochłowice. More detailed data on the timing and frequency of using DSSWR should also be included. Second, PA was calculated using a self-assessment method; therefore, a subjective interpretation of the questions may have influenced the results. In addition, the IPAQ-SF may have overestimated the PA level [58]. The %BF was estimated using an Omron BF-306 body composition monitor. According to Jensky-Squires et al. [59], the %BF output from Omron devices should be interpreted with caution.

The main strength of this paper was the inclusion of sociodemographic and anthropometric (BMI, WHR and %BF) factors and PA in explaining DSSWR use. Previous studies have predominantly examined the association between single factors and DSSWR use. Another strength of this study was the inclusion of a large study population. The direct measurements of weight and height, and waist and hip circumference used in our research allowed for accurate BMI and WHR calculations. Despite some limitations concerning the measurement of %BF using bioimpedance, the use of an objective method with such a large sample may be considered to be a strength of the study.

## Conclusion

It seems that primarily financial status and sex, but also age, education and level of PA, have significance in DSSWR use in adults and may be considered when developing appropriate strategies for body weight management and health promotion. This should allow public authorities at various levels to coordinate their sectoral activities and implement a cohesive health policy that also includes PA.

Due to the widespread use of DSSWR, these findings appear relevant not only for nutrition specialists, but also for health promoters and employees of sports and fitness clubs, who should pay particular attention to physically active patients/clients with the listed sociodemographic characteristics, and increase these patients’/clients’ awareness of the risks involved in DSSWR use.

## Data Availability

The datasets used and analysed during the current study are available from the corresponding author on reasonable request.

## Abbreviations

%BF: Percent body fat
BMI: Body mass index
DSSWR: Dietary supplements supporting weight reduction
PA: Physical activity
WHR: Waist-hip ratio
